# Primary gastric squamous cell carcinoma presenting as a large submucosal mass: A case report and literature review

**DOI:** 10.1097/MD.0000000000022125

**Published:** 2020-09-04

**Authors:** Lei Gao, Xiaolong Tang, Hui Qu, Qingsi He, Guorui Sun, Jingbo Shi, Jianhong Ye, Yahang Liang

**Affiliations:** a Qilu Medical College of Shandong University; b Department of General Surgery, Qilu Hospital of Shandong University, Jinan China.

**Keywords:** diagnosis, gastric neoplasms, gastrointestinal stromal tumor, prognosis, squamous cell carcinoma

## Abstract

**Rationale:**

Primary gastric squamous cell carcinoma (SCC) is rarely encountered clinically. SCC, which presents as a submucosal tumor, is even rarer. Without the support of pathological evidence, it is difficult to make a correct preoperative diagnosis. Due to limited clinical data, the pathogenesis and treatment of gastric SCC remain unclear.

**Patient concerns:**

A 69-year-old man was admitted to our hospital with unexplained weight loss. Endoscopy revealed a submucosal mass without any ulcer on its surface located on the body of the stomach. The results of 2 gastroscopic mucosal biopsies were chronic inflammation.

**Diagnoses:**

The clinical diagnosis by computed tomography (CT) and gastroscopy was gastrointestinal stromal tumor (GIST) preoperatively. The postoperative pathological examination demonstrated this tumor as moderately differentiated SCC.

**Interventions:**

Total gastrectomy, distal pancreatectomy, and splenectomy were performed.

**Outcomes:**

The patient was discharged 7 days after the surgery without any complications. The follow-up CT scan showed no evidence of metastatic disease 6 months after surgery.

**Lessons:**

Large primary gastric SCC could present as a submucosal mass. Gastroscopic mucosal biopsy may not be able to get tumor tissue due to inflammatory reaction.

## 1 Introduction

Primary gastric squamous cell carcinoma (GSCC) is a rare malignancy, only accounts for 0.2% of all gastric neoplasms.^[1]^ Less than 100 primary GSCCs have been reported so far.^[2–4]^ In compliance with the Japanese Society of Gastric Cancer, primary GSCC was defined as the squamous cell carcinoma (SCC) originating entirely from the stomach without any adenocarcinoma components.^[5]^ The symptoms of primary GSCC are similar to those of gastric adenocarcinoma such as abdominal pain and weight loss. However, patients with primary GSCC usually had a poorer prognosis than those with adenocarcinoma.^[6]^ Due to limited clinical data, the standard treatment for primary GSCC had not come to an agreement. It is currently believed that radical surgery can benefit patients.

The majority of primary GSCCs reported presented as ulcer lesions. We experienced a case of large primary GSCC presenting as a large submucosal tumor, which was rarely reported. Without pathological evidence, we misdiagnosed the tumor as gastrointestinal stromal tumor (GIST) preoperatively. This study provided an extremely rare case and reviewed 25 cases of primary GSCC published, which could provide help for the diagnosis and treatment of primary GSCC.

## 2 Case presentation

**Figure 1:**
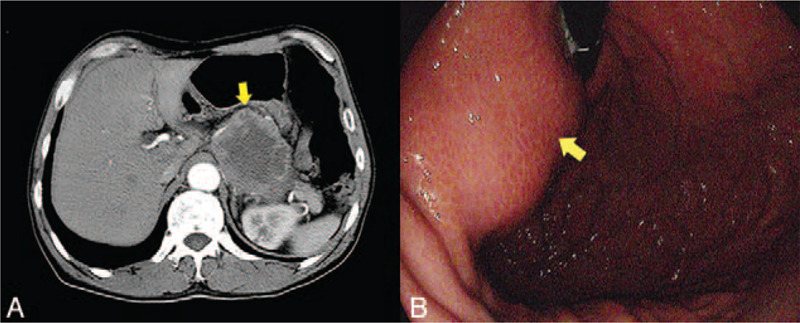
Images of CT and gastroscopy. Contrast-enhanced CT showed a large exophytic mass (yellow arrow) on the body of the stomach (A). The gastroscopy indicated a submucosal mass (yellow arrow) (B).

**Figure 2:**
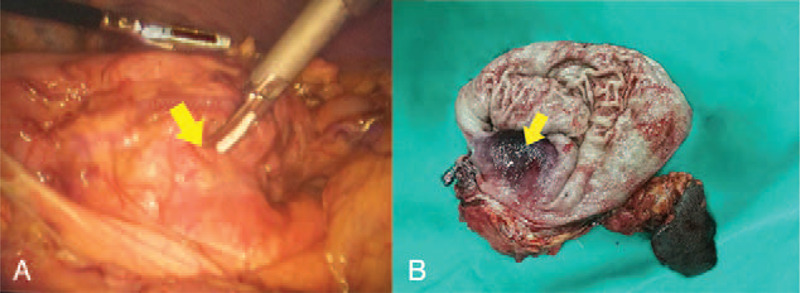
Images of laparoscopic exploration and the specimen. Laparoscopy revealed a large exophytic mass (yellow arrow) originating from the stomach and invading into the pancreas (A). Resected specimen showed the submucosal tumor (yellow arrow) (B).

**Figure 3:**
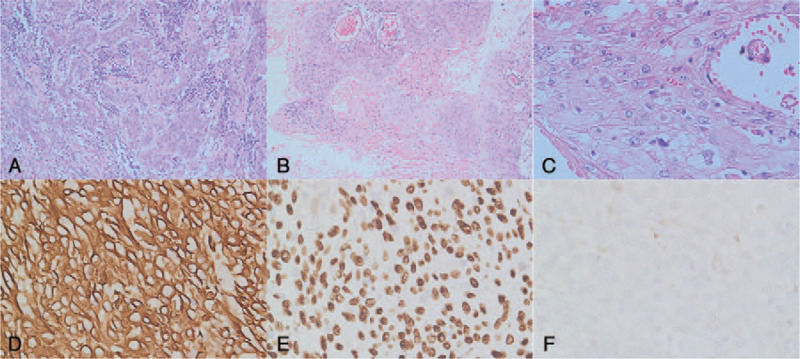
Histological and immunohistochemical (H&E) examination. H&E examination showed a moderately differentiated SCC (A, ×20 magnification) with keratin pearls (B, ×10 magnification) and intracellular bridges (C, ×40 magnification). Immunohistochemical staining displayed that the tumor cells were positive for CK 5/6 (D, ×40 magnification) and P63 (E, ×40 magnification) and negative for CD117 (F, ×40 magnification).

A 69-year-old man came for consultation due to an unexplained weight loss of 3 kg in 2 months. He denied other symptoms such as anorexia, nausea, vomiting, fatigue, or melena. The laboratory tests were normal. Computed tomography (CT) showed a heterogeneously enhanced tumor mass (Fig. 1A) located between the pancreas and the stomach, and no evidence of metastasis was found in other parts of the body. Endoscopy revealed a submucosal mass without any ulcer on its surface located on the body of the stomach (Fig. 1B). The first gastroscopic mucosal biopsy showed chronic inflammation, so we performed the second biopsy and the result were consistent with the first. The third mucosal biopsy was refused by the patient. The clinical diagnosis by CT and gastroscopy was GIST. Therefore, laparoscopic exploration was performed, which revealed a large and exophytic mass originating from the posterior wall of the stomach and invading the pancreas and splenic vessels (Fig. 2). The tumor was completely dissected using total gastrectomy, distal pancreatectomy, and splenectomy. The intraoperative rapid frozen pathology found SCC. The postoperative pathological examination found moderately differentiated SCC in the resected specimen (Fig. 3A-C). No metastasis was observed in the 19 dissected lymph nodes. Immunohistochemistry markers were positive for P63 and cytokeratin (CK) 5/6 and negative for the cluster of differentiation (CD) 117 (Fig. 3D-F). No evidence of SCC in other organs was reported. Therefore, the postoperative diagnosis was primary GSCC. According to the Eighth Edition AJCC Cancer Staging Manual, it was diagnosed as T4bN0M0, pStage IIIA. The patient was discharged 7 days after surgery without any complications and referred to the medical oncology to receive adjuvant chemotherapy, but he refused. The follow-up CT scan showed no evidence of metastatic disease 6 months after the surgery.

## 3 Discussion and conclusions

GSCC is characterized by keratin pearls, mosaic cell arrangement, intercellular bridges, and a high concentration of sulfhydryl and/or disulfide groups.^[7]^ Most gastric SCCs are located on the esophagogastric junction. Primary GSCC is rarely encountered clinically. The Japanese Society of Gastric Cancer provided the concept of “primary”.^[5]^ The tumor was not located on cardia and did not invade into the esophagus. No evidence of SCC in any other organs was reported. In our cases, endoscopy showed that the tumor was located on the body of the stomach and did not invade the esophagus. Thoracic, abdominal, and pelvic of contrast-enhanced CT showed no evidence of metastatic lesions. Therefore, the diagnosis was primary GSCC.

The pathogenesis of primary GSCC remains unclear. Several theories about the origin of primary GSCC have been proposed, including squamous differentiation followed by adenocarcinoma, squamous metaplasia of gastric mucosa, multipotent capability of stem cells, nests of ectopic squamous epithelium in the gastric mucosa, and malignant transformation of gastric vascular endothelial cells.^[8,9]^

Fewer than 100 cases of primary GSCC have been reported.^[2]^ However, it is difficult to detect accurate clinical features of this disease. A total of 25 case reports in the English literature published in the last 10 years were reviewed.^[2–4,8–29]^ The clinical data are shown in Table 1. The mean age at the onset was 64 years, and the male-to-female ratio was 19:6. The most common symptoms were abdominal pain (13/25) and weight loss (12/25). Most tumors exceed 5 cm and were diagnosed in an advanced stage.

**Table 1:**
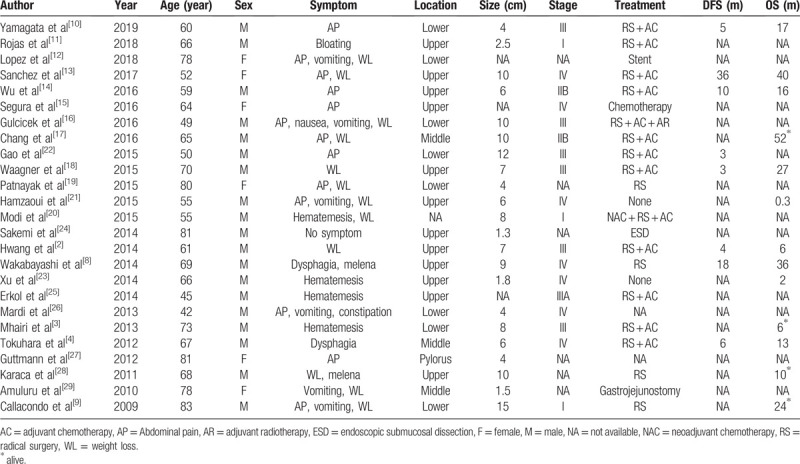
Case reports of primary gastric squamous cell carcinoma published in the last 10 years.

GSCC presenting as a submucosal mass occurred in only 1 of 25 cases.^[2]^ It should be differentiated from other neoplasms, including GIST, carcinoids, melanomas, lymphomas, and leiomyosarcomas. The most common submucosal tumors are GIST. The CT scan for GSCC usually shows a heterogeneously enhanced mass.^[13,14,17]^ The same performance is reflected on huge GIST due to necrosis, hemorrhage, or degenerative components.^[30]^ Gastroscopic mucosal biopsy could help in identifying the submucosal tumors. However, a preoperative biopsy was not recommended for the tumors that were clinically highly suspected of GIST and could be completely removed.^[31]^ We support the idea of preoperative biopsy, because the preoperative diagnosis is important to decide surgical method. Endoscopic ultrasound-fine needle autopsy should be considered when no abnormalities are found in the first biopsy. CK 5/6, P63, and CD117 are immunohistochemical markers that help distinguish between SCC and GIST.^[32,33]^ Therefore, large primary GSCC, which resembles a submucosal mass, could be misdiagnosed as GIST in the absence of pathological evidence.

The prognosis of gastric SCC was dismal.^[1,34]^ The study by Caixia Dong et al analyzed the prognostic characteristics of primary GSCC.^[1]^ It was reported that 47.2% of patients were diagnosed at stage IV. The median survival for gastric adenocarcinoma in this study was 19 months, while the primary GSCC was only 8 months. The 5-year overall survival for primary GSCC was 32.7%, respectively, and patients in stage I, II, III, and IV was 80.0%, 67.5%, 39.7%, and 6.0%, respectively. More importantly, their study indicated the prognosis of the surgical group and the non-surgical group was significantly different. The median survival for the surgery group was 133 months, and the 5-year survival rate was 59.2%. However, the median survival for non-surgery group was 2 months, and the 5-year survival rate was 17.4%.

Phases 2 and 3 randomized controlled trial proved that chemoradiotherapy followed by surgery improved the survival of patients with SCC in the esophagus and esophagogastric junction compared with surgery alone.^[35,36]^ Due to limited data, no guideline exists for primary GSCC. Surgery combined with chemotherapy is the most common treatment.

The majority of primary GSCC presented as ulcer lesion. By endoscopy and biopsy, it was not hard to make a correct diagnosis preoperatively. Our case indicated that primary GSCC could present as submucosal tumor. The extremely rare case, combined with the inflammatory reaction around the tumor, made us not get the correct diagnosis until the postoperative histopathological examination was completed. When it was difficult to obtain lesion tissue, multi-point sampling should be considered or with the help of endoscopic ultrasonography. Preoperative diagnosis is important to decide surgical method. Therefore, repeated biopsy is useful for diagnosis of submucosal tumor.

In conclusion, a rare case of primary GSCC presenting as a submucosal tumor was reported, and 25 case reports in the English literature published in the last 10 years were reviewed. Primary GSCC could present as a submucosal tumor, and gastroscopic mucosal biopsy may not be able to get tumor tissue due to inflammatory reaction. Surgery combined with chemotherapy is the most common treatment. The prognosis of primary GSCC is poor.

## Author contributions

**Conceptualization:** Hui Qu, Lei Gao, Xiaolong Tang.

**Formal analysis:** Lei Gao.

**Investigation:** Lei Gao, Xiaolong Tang, Guorui Sun.

**Methodology:** Hui Qu, Qingsi He.

**Project administration:** Hui Qu, Qingsi He.

**Resources:** Guorui Sun, Jingbo Shi, Jianhong Ye, Yahang Liang.

**Supervision:** Hui Qu, Qingsi He, Guorui Sun.

**Visualization:** Hui Qu, Qingsi He, Guorui Sun.

**Writing – original draft:** Lei Gao, Xiaolong Tang.

**Writing – review & editing:** Lei Gao, Xiaolong Tang, Hui Qu.

## References

[1] Dong C Jiang M Tan Y The clinicopathological features and prognostic factors of gastric squamous cell carcinoma. Medicine (Baltimore) 2016;95:e4720. 27559983 10.1097/MD.0000000000004720 PMC5400350

[2] Hwang SH Lee JH Kim K Primary squamous cell carcinoma of the stomach: a case report. Oncol Lett 2014;8:2122 4. 25289093 10.3892/ol.2014.2492 PMC4186502

[3] Little M Munipalle PC Viswanath YK Primary squamous cell carcinoma of the stomach: a rare entity. BMJ Case Rep 2013;2013. 10.1136/bcr-2013-009706 PMC3669995 23709554

[4] Tokuhara K Nakano T Inoue K Primary squamous cell carcinoma in the gastric remnant. Surg Today 2012;42:666 9. 22350299 10.1007/s00595-012-0144-6

[5] Japanese Gastric Cancer A. Japanese classification of gastric carcinoma: 3rd English edition. Gastric Cancer 2011;14:101 12. 21573743 10.1007/s10120-011-0041-5

[6] Meng Y Zhang J Wang H Poorer prognosis in patients with advanced gastric squamous cell carcinoma compared with adenocarcinoma of the stomach: case report. Medicine (Baltimore) 2017;96:e9224. 29390350 10.1097/MD.0000000000009224 PMC5815762

[7] Boswell JT Helwig EB Squamous cell carcinoma and adenoacanthoma of the stomach. a clinicopathologic study. Cancer 1965;18:181 92. 14254074 10.1002/1097-0142(196502)18:2<181::aid-cncr2820180209>3.0.co;2-3

[8] Wakabayashi H Matsutani T Fujita I A rare case of primary squamous cell carcinoma of the stomach and a review of the 56 cases reported in Japan. J Gastric Cancer 2014;14:58 62. 24765539 10.5230/jgc.2014.14.1.58 PMC3996251

[9] Callacondo D Ganoza-Salas A Anicama-Lima W Primary squamous cell carcinoma of the stomach with paraneoplastic leukocytosis: a case report and review of literature. Hum Pathol 2009;40:1494 8. 19467693 10.1016/j.humpath.2009.02.014

[10] Yamagata Y Saito K Ban S The origin of p40-negative and CDX2-positive primary squamous cell carcinoma of the stomach: case report. World J Surg Oncol 2019;17:53. 30890174 10.1186/s12957-019-1594-8 PMC6425685

[11] Guzman Rojas P Parikh J Vishnubhotla P Primary gastric squamous cell carcinoma. Cureus 2018;10:e2389. 29850384 10.7759/cureus.2389 PMC5973482

[12] Fraile Lopez M Mendoza Pacas GE Fernandez Cadenas F Primary squamous cell carcinoma of the stomach: a rare entity. Rev Esp Enferm Dig 2018;111. 10.17235/reed.2018.5900/2018 30511579

[13] Gonzalez-Sanchez JA Viton R Collantes E Primary squamous cell carcinoma of the stomach. Clin Med Insights Oncol 2017;11:1179554916686076. 28469507 10.1177/1179554916686076 PMC5395270

[14] Wu XD Zhou Y Fan RG Primary squamous cell carcinoma of the stomach presenting as a huge retroperitoneal tumor: a case report. Rev Esp Enferm Dig 2016;108:283 4. 26181433 10.17235/reed.2015.3795/2015

[15] Segura S Pender J Dodge J Primary squamous cell carcinoma of the stomach: a case report and review of the literature. Conn Med 2016;80:209 12. 27265923

[16] Gulcicek OB Solmaz A Ozdogan K Primary squamous cell carcinoma of the stomach. Ulus Cerrahi Derg 2016;32:221 3. 27528817 10.5152/UCD.2015.2811 PMC4970785

[17] Chang YS Kim MS Kim DH Primary squamous cell carcinoma of the remnant stomach after subtotal gastrectomy. J Gastric Cancer 2016;16:120 4. 27433399 10.5230/jgc.2016.16.2.120 PMC4944001

[18] von Waagner W Wang Z Picon AI A rare case of a primary squamous cell carcinoma of the stomach presenting as a submucosal mass. Case Rep Surg 2015;2015:482342. 26185704 10.1155/2015/482342 PMC4491394

[19] Patnayak R Reddy V Radhakrishnan Primary squamous cell carcinoma of stomach: a rare entity - case report and brief review of literature. J Surg Tech Case Rep 2015;7:45 7. 27512553 10.4103/2006-8808.185656 PMC4966205

[20] Modi Y Shaaban H Parikh N Primary pure squamous cell carcinoma of the stomach treated with neoadjuvant chemotherapy and surgical resection. Indian J Cancer 2015;52:145. 26838004 10.4103/0019-509X.175570

[21] Hamzaoui L Bouassida M Kilani H Metastatic squamous cell carcinoma of the stomach. J Clin Diagn Res 2015;9:OD05 06. 10.7860/JCDR/2015/14527.6720 PMC4668455 26673808

[22] Gao S Chen D Huang L Primary squamous cell carcinoma of the stomach: a case report and literature review. Int J Clin Exp Pathol 2015;8:9667 71. 26464735 PMC4583967

[23] Xu F Feng GS Wang ZJ Synchronous double cancers of colonic large cell neuroendocrine carcinoma and gastric squamous-cell carcinoma: a case report and review of literature. Int J Clin Exp Pathol 2014;7:5177 80. 25197393 PMC4152083

[24] Sakemi R So S Morimitsu Y Endoscopic submucosal dissection of squamous cell carcinoma in the upper stomach 5 years after chemoradiotherapy for adenocarcinoma. Clin J Gastroenterol 2014;7:310 5. 26185878 10.1007/s12328-014-0503-5

[25] Erkol B Tilki M Comunoglu N Neuroendocrine/squamous gastric collision tumor: a rare entity. Turk J Gastroenterol 2014;25: Suppl 1: 282 3. 25910342 10.5152/tjg.2014.5425

[26] Mardi K Mahajan V Sharma S Primary squamous cell carcinoma of stomach: a rare case report. South Asian J Cancer 2013;2:199. 10.4103/2278-330X.119897 PMC3889029 24455626

[27] Guttmann S Fromer N Shamah S A case of two primary gastric malignancies: adenocarcinoma and squamous cell carcinoma of the stomach. Gastrointest Endosc 2012;75:1113 4. 21802677 10.1016/j.gie.2011.05.037

[28] Karaca G Pekcici MR Ozer H Primary squamous cell carcinoma of the stomach in a 68-years-old man. Geriatr Gerontol Int 2011;11:119 20. 21166967 10.1111/j.1447-0594.2010.00642.x

[29] Amuluru K Gupta H Primary squamous cell carcinoma of the stomach: a case report. J Gastrointest Cancer 2010;41:24 6. 19960279 10.1007/s12029-009-9097-4

[30] Kim JS Kim HJ Park SH Computed tomography features and predictive findings of ruptured gastrointestinal stromal tumours. Eur Radiol 2017;27:2583 90. 27761711 10.1007/s00330-016-4515-z

[31] Sanchez-Hidalgo JM Duran-Martinez M Molero-Payan R Gastrointestinal stromal tumors: a multidisciplinary challenge. World J Gastroenterol 2018;24:1925 41. 29760538 10.3748/wjg.v24.i18.1925 PMC5949708

[32] DiMaio MA Kwok S Montgomery KD Immunohistochemical panel for distinguishing esophageal adenocarcinoma from squamous cell carcinoma: a combination of p63, cytokeratin 5/6, MUC5AC, and anterior gradient homolog 2 allows optimal subtyping. Hum Pathol 2012;43:1799 807. 22748473 10.1016/j.humpath.2012.03.019 PMC3465493

[33] Wong NA Gastrointestinal stromal tumours--an update for histopathologists. Histopathology 2011;59:807 21. 21668468 10.1111/j.1365-2559.2011.03812.x

[34] Akce M Jiang R Alese OB Gastric squamous cell carcinoma and gastric adenosquamous carcinoma, clinical features and outcomes of rare clinical entities: a National Cancer Database (NCDB) analysis. J Gastrointest Oncol 2019;10:85 94. 30788163 10.21037/jgo.2018.10.06 PMC6351299

[35] van Meerten E Muller K Tilanus HW Neoadjuvant concurrent chemoradiation with weekly paclitaxel and carboplatin for patients with oesophageal cancer: a phase II study. Br J Cancer 2006;94:1389 94. 16670722 10.1038/sj.bjc.6603134 PMC2361286

[36] van Hagen P Hulshof MC van Lanschot JJ Preoperative chemoradiotherapy for esophageal or junctional cancer. N Engl J Med 2012;366:2074 84. 22646630 10.1056/NEJMoa1112088

